# Opinion: the Future of Electrical Impedance Tomography

**DOI:** 10.2478/joeb-2022-0001

**Published:** 2022-03-31

**Authors:** Kirill Aristovich

**Affiliations:** 1 Dept. of Med. Phys. & Biomedical Eng., Faculty of Engineering Science, University College London, London United Kingdom

Traditionally, medical electrical impedance tomography (EIT) [1] was viewed as a portable and inexpensive technique, which would be mass-produced and have the potential to be a good diagnostic tool in multitudes of clinical applications. Unfortunately, this has not happened. This is for various reasons, the primary being that it is very easy to do, but very hard to do it right. It transpires there are multiple stages where things can and will go wrong, and one has to excel in all of them to achieve a good result.

First of all, a simple shift from serial single 4-electrode impedance measurement to multiple electrodes is hard, because one suddenly needs to consider stray capacitance, cross-talk, electrode impedance and electronics mismatch, which induces uneven noise distributions and an array of other problems. That is why, thus far, single-injection-pair systems, that switch between the pairs so that the electronic components are identical for all injections, dominate the field in practice over the multiple-inject systems where everything needs to be just perfect otherwise would not work.

Secondly, there is an image reconstruction problem, of which there are numerous flavors ranging from simple back-projection to advanced machine learning, with various degrees of applicability in real life. These two obstacles are challenging but might have been tractable as the geophysics community has proven in use of electrical resistance tomography (ERT) as a routine survey method. However, in biomedical EIT there are unfortunately two other distinct challenges.

The third one is the subject of the human body being a complex system of tissues comprising layered resistive and underlying conductive materials with, in the majority of cases, an anisotropic conductivity.

And the last challenge, which is probably predominant, is the human error of the operator itself. One has to place the electrode perfectly, ensuring the contact is good, acquire the data checking for no interference, recognize and remove artefacts, of which there are many, and interpret the data correctly and objectively eliminating so common cases of “garbage in -> garbage out” mistakenly observed as genuine impedance images.

**Figure 1:**
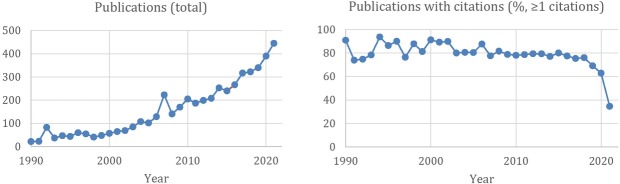
The total number of publications and citations with the phrase “Electrical Impedance Tomography” in the title or abstract raised exponentially each year (left), while the number of publications with the same criteria but having at least one citation, stayed the same and dropped slightly over the past four years (right) [1].

Overall, a fault tree analysis shows that the probability of success is very low because each of the above issues is a single point of failure, bringing the respectful per cent success rate in every stage down to below 50% overall as probabilities multiply, and to achieve good reliability one needs more than 95% on every stage. I believe that it is because of this the number of publications with citations in EIT remains the same across the years, in fact dropping over the last four years, while the total number of publications together with the total number of citations rises exponentially (see figure 1). It looks like good trustworthy data is only coming from a few very specialized groups which maintain the culture, the knowledge of the main drawbacks, and can ensure all stages of the technique are achieved with 95% accuracy, which in turn means that the said groups should have experts in electronics, mathematics, physiology, and medicine at the same time.

All the above may be summarized in the simple statement: it is very easy to do EIT, but it is very hard to do it properly. As a result, EIT has become a highly specialized technique applied for a very particular set of problems where there are no other alternatives. Examples include imaging functional activity in brains and nerves, imaging particular cancer types, and differentiating stroke.

The only exception is lung imaging, but arguably this is the simplest application of EIT as it is performed in time difference mode with the infinite conductivity contrast thus partially eliminating hardware and reconstruction problems. Even then the actual clinical spread of the technique has been limited.

There can only be two real practical solutions to this problem. First, we can increase the probability of success for each of the above single points of failure and make sure that the solutions are accessible for everyone. Recently some progress had been achieved with the publications of open-source hardware, designs, software, and protocols aimed at modularity and unification, and at the same time lowering the costs and increasing the accuracy of EIT systems production [3, 4, 5, 6]. This renders it possible to unify the technique and provide the solution which potentially would fit all applications, after which it can be mass-produced on a massive scale which would neglect the complexity of the design. However, there seems to be no unique formula for the parameters which fits all the applications, and sometimes the requirements contradict each other: A good example is imaging in the brain where for functional imaging the frequency should be tuned to a particular low kilohertz range and optimized with respect to the particular bandwidth, while for imaging of stroke a well-balanced spectrum is required. This imbalance of optimal parameters might obviate the idea of a mass-produced fit-them-all technique which then leaves only a handful of applications. Lung imaging might be one of them, and the solution to a wider clinical spread might be just in cost-benefit analysis, so it will settle out with time when the market will establish fully and clinical chest EIT belts will become a cheap consumable.

The second, and slightly more promising trend is that the number of above single points of failure could be reduced by fusion with other techniques and physical paradigms. Among the recent examples of this are the rapid developments of Magnetic Resonance EIT (MREIT) [1], which can potentially add an array of useful diagnostic measures on top of traditional MRI and fMRI. There is also Acousto-Electric Tomography (AET) [1] which is based on the images produced by the electroacoustic effect when conductivity is changed by the deformation of ion-rich tissues. AET can potentially have the same resolution as ultrasonic imaging and holds a promise to resolve the images of absolute conductivity, thus becoming a widespread technique for imaging any human organ in the future.

Further work has been done in other directions, but I think the general trend is clear: EIT is unlikely to become a cheap and cheerful fit-them-all technique used for all possible imaging tasks, like a cheap replacement of CT or MRI, and will continue to evolve and be tuned for niche specialized cases. However, in those cases, it can be disruptive and life changing as there are no alternatives, even theoretical, of obtaining this crucial medical information which is then directly employed for patient treatment.
